# Phylogeography and Genetic Diversity of *Rana kukunoris* on the Northeast Qinghai-Xizang Plateau: Insights from Mitochondrial Cytochrome b Gene

**DOI:** 10.3390/ani16071013

**Published:** 2026-03-26

**Authors:** Fuhao Zhang, Bao Dong, Ying Zhao, Wanting Wang, Yanfeng He, Xuze Zhang

**Affiliations:** 1 School of Ecological Environment and Resources, Qinghai Minzu University, Xining 810007, China; 2 School of Pharmacy, Qinghai Minzu University, Xining 810007, China; 3 Qinghai Provincial Key Laboratory of High-Value Utilization of Characteristic Economic Plants, Xining 810007, China; 4 Qinghai Provincial Biotechnology and Analytical Test Key Laboratory, Xining 810007, China

**Keywords:** *Rana kukunoris*, cytochrome b, genetic diversity, gene flow, phylogenetic relationships

## Abstract

*Rana kukunoris* is an amphibian species endemic to the Qinghai-Xizang Plateau and adapted to high-elevation environments. To clarify its genetic diversity and phylogeographic structure, mitochondrial cytochrome b (*Cytb*) sequences from multiple localities across the northeastern plateau were analyzed. The results indicate moderate haplotype diversity coupled with low nucleotide variation, suggesting a demographic history shaped by historical bottlenecks and limited expansion. Furthermore, population genetic analyses reveal restricted gene flow and notable genetic differentiation among geographically separated localities. Phylogenetic reconstruction supports the presence of two major lineages, corresponding to northern and southern groups divided by an elevational boundary at approximately 3200 m. Historical demographic inference further suggests regionally distinct population dynamics rather than a pronounced, synchronous expansion. Overall, these findings demonstrate that plateau topography and Quaternary climatic oscillations have played key roles in shaping the evolutionary history and current genetic structure of *R. kukunoris*.

## Simple Summary

*Rana kukunoris* is a high-altitude amphibian endemic to the Qinghai-Xizang Plateau. To clarify its genetic structure and evolutionary history, we analyzed mitochondrial *Cytb* sequences from 105 individuals collected across 14 localities in the northeastern plateau. Twenty-one haplotypes were identified, and populations exhibited moderate haplotype diversity but low nucleotide diversity, a pattern consistent with historical bottlenecks followed by limited expansion. Phylogenetic and haplotype network analyses revealed two major lineages (northern and southern) separated by an elevational boundary at approximately 3200 m. Population genetic analyses indicated restricted gene flow and significant differentiation among several geographically isolated populations. Historical effective population size reconstructions suggested regionally distinct demographic trajectories rather than a pronounced synchronous expansion. Together, these results indicate that topographic barriers and Quaternary climatic oscillations jointly shaped the current genetic structure of *R. kukunoris* and provide a genetic basis for its conservation management.

## 1. Introduction

*Rana kukunoris* (Nikolsky, 1918) [1], belonging to the order Anura, family Ranidae, and genus *Rana*, inhabits a variety of aquatic habitats and adjacent moist environments at elevations ranging from 2000 to 4200 m above sea level. It is primarily distributed in eastern Xizang, most areas of Qinghai, the mountainous and plateau regions of northwestern Sichuan, and western Gansu [2]. This species is endemic to China [3]. The dorsal surface is typically earthy yellow, grayish-brown, or reddish-brown. Males generally exhibit a pale yellow ventral surface, whereas females possess a brownish-red or orange-red abdomen. Distinct red or black blotches occur on the flanks, and the limbs bear dark transverse bands. Dark brown or orange-red spots are present on the tubercles and associated structures, and the dorsolateral folds are reddish-brown. Adults measure approximately 4–6 cm in body length. The dorsolateral folds are curved and extend above the tympanum. When the hindlimb is stretched forward along the body, the tibiotarsal articulation reaches beyond the eye or nostril. The hindlimb length is approximately 185% of the body length, and the webbing between the outer three toes extends to about two-thirds of the toe length. A distinct triangular black marking is present near the tympanum. Males possess two well-developed nuptial pads at the base of the first finger and a pair of internal vocal sacs beneath the throat [2]. Compared with *Rana chensinensis*, *R. kukunoris*, which inhabits higher-altitude regions, generally exhibits a smaller body size, relatively shorter hindlimbs, a head length shorter than head width, darker overall coloration, rougher dorsal skin, and an orange-red ventral surface in females [4]. Further histological investigations have demonstrated significant inter-regional differences in dermal thickness, gland distribution, and chromatophore abundance across different body parts of *R. kukunoris* [5]. Moreover, the observed color polymorphism in this species is closely associated with differentiated adaptive strategies for coping with ultraviolet radiation [6]. In 1918, Nikolsky described *Rana amurensis kukunoris* as a new subspecies of *R. amurensis* based on specimens collected from Qinghai Lake [1]. Subsequently, Boring reassigned this taxon to *Rana temporaria chensinensis*, treating it as a subspecies of *R. chensinensis* [7]. Through taxonomic research on *R. kukunoris*, Xie et al. proposed that the various localities related to *R. chensinensis* in northwestern China comprise two valid species, namely *R. chensinensis* and *R. kukunoris* distributed in the Qinghai-Xizang Plateau (QTP) region [4]. This conclusion was further corroborated by Jiang et al. based on analyses of both the single 12S marker and the combined 12S-*Cytb* dataset [8,9]. Phylogenetic reconstruction using complete mitochondrial genome sequences revealed a close evolutionary affinity between *R. kukunoris* and *R. chensinensis* [10,11]. Both species are inferred to have originated from a common low-altitude ancestor, and divergence time estimates suggest that the speciation of *R. kukunoris* coincided with the uplift of the Qinghai-Xizang Plateau approximately 7.8 million years ago, highlighting the potential role of geological events in shaping lineage diversification [12].

The mitochondrial genome is characterized by a simple structure, absence of recombination, maternal inheritance, and a relatively high evolutionary rate, making it a crucial genetic marker for studying species phylogeny and population genetic structure across diverse taxa [13]. Indeed, mitochondrial DNA is among the most widely used molecular markers in evolutionary biology, providing robust evidence for evolutionary relationships in a wide range of organisms, from invertebrates to avian species [14,15]. Furthermore, the D-loop region and protein-coding sequences, such as the *Cytb* gene, have been frequently utilized to resolve complex evolutionary histories in various mammalian models and high-altitude species [16,17,18]. Building upon this universal methodological foundation, these markers have been successfully adopted to clarify phylogenetic relationships within the genus *Rana*, where the mitochondrial *Cytb* gene is widely regarded as a highly effective and informative marker for evaluating genetic variation and evolutionary dynamics [19]. Yang et al. determined *Cytb* sequences of *Rana* and *Pelophylax* species in China and showed that *R. chensinensis* has the closest genetic relationship with *R. amurensis* and the most distant relationship with *Rana huanrenensis* [20]. Zhou et al. explored the validity of various species in the genus *Rana* and their phylogenetic relationships based on mitochondrial *Cytb* gene sequences of *R. chensinensis*; the results supported the monophyly of the *R. chensinensis* species group, *Rana longicrus* species group, and *R. amurensis* species group [21]. Dong et al. amplified the *Cytb* sequence of *Rana dybowskii* using PCR technology, thereby establishing a molecular identification method for *Rana* species and the origin of *Oviductus Ranae* [22]. Zhou et al. analyzed mitochondrial and nuclear gene sequences of the *R. chensinensis* complex [12]. The results indicated that the four major clades of the *R. chensinensis* complex include *R. huanrenensis*, the Qinling localities of *R. chensinensis* (type locality), *R. kukunoris*, and the Loess Plateau localities of *R. chensinensis*. Haplotype nesting was observed between *R. chensinensis* from the Qinling region and *R. kukunoris*, and gene flow exists within the *R. chensinensis* complex [12]. Population differentiation was also detected in *R. kukunoris*. However, phylogenetic relationships within the genus *Rana* remain complex, and previous studies have mainly focused on specific species groups. A more comprehensive phylogenetic framework incorporating newly described species remains to be established [21].

Genetic diversity provides the foundation for species adaptation to environmental change and the maintenance of biodiversity. Furthermore, it plays an essential role in sustaining population reproduction, habitat adaptation, and disease resistance [23]. Amphibians generally have limited dispersal ability and are highly susceptible to geographic isolation, which can promote divergence and evolutionary differentiation, making them ideal model organisms for molecular systematics and phylogeographic studies [24]. Therefore, analyzing the genetic diversity of *R. kukunoris* is crucial for understanding its migration patterns, population differentiation, and conservation during adaptation to plateau environments. In this study, complete *Cytb* sequences were used to assess the genetic diversity and phylogenetic relationships of 14 localities of *R. kukunoris*, providing a theoretical basis for the conservation of its genetic resources.

## 2. Materials and Methods

### 2.1. Sample Collection

**Figure 1:**
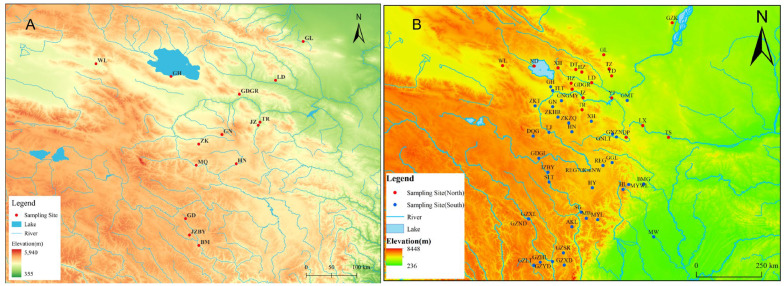
Sampling site distribution of *R. kukunoris.* **Note:** In (**A**), GL, LD, JZ, GN, QHH, WL, TR, ZK, HN, GDGR, MQ, JZBY, BM, GD represent Gulang of Gansu, Ledu of Qinghai, Jianzha of Qinghai, Guinan of Qinghai, Gonghe of Qinghai, Wulan of Qinghai, Tongren of Qinghai, Zeku of Qinghai, Henan of Qinghai, Guide Garang of Qinghai, Maqin of Qinghai, Jiuzhi baiyu of Qinghai, Banma of Qinghai, Gande of Qinghai. In (**B**), red represents the northern clade and blue represents the southern clade.

*R. kukunoris* were collected from 14 regions: Gulang (GL) in Gansu Province, and Guinan (GN), Wulan (WL), Ledu (LD), Jianzha (JZ), southern Gonghe (GH), Tongren (TR), Zeku (ZK), Henan (HN), Guide (GDGR), Baiyu in Jiuzhi (JZBY), Banma (BM), and Gande (GD) in Qinghai Province (Figure 1). Specimens were identified in the field based on morphological diagnostic characters described in the literature, including body size, dorsal coloration, dorsolateral fold features, and tympanum morphology. All samples collected from the wild were preserved in 95% ethanol and subsequently transported to the laboratory for storage. Subsequently, the specimens were dissected in the laboratory to harvest muscle tissue for downstream mitochondrial DNA (mtDNA) extraction. Detailed sampling information is provided in Table 1.

### 2.2. Extraction of Mitochondrial DNA

Genomic DNA was extracted from the muscle tissue of *R. kukunoris* using the phenol-chloroform extraction method [25]. DNA integrity and fragment size were verified by 1% agarose gel electrophoresis with a commercial DNA molecular weight marker. DNA quality and concentration were further assessed using a NanoDrop spectrophotometer. Qualified DNA samples were subsequently stored at −20 °C for further analyses.

### 2.3. PCR Amplification

Based on the mitochondrial DNA *Cytb* sequence of *R. kukunoris* in the NCBI database (GenBank accession number: MZ043820), PCR primers were designed using Primer-BLAST 5.0 for amplifying the *Cytb* sequence. The primer sequences were as follows: forward primer *Cytb*F (5′-ACAATACGTAAATCTCACC-3′) and reverse primer *Cytb*R (5′-AAGTTTATTTTCTAGGAGACC-3′), which were synthesized by Sangon Biotech (Shanghai) Co., Ltd. (Shanghai, China).

The total volume of the PCR reaction was 25 μL, and the reaction system was as follows: 12.5 μL of 2 × San Taq PCR Mix (containing blue dye), 1 μL of DNA template, 0.5 μL each of the forward and reverse primers, and finally 10.5 μL of double-distilled water to make up the volume. The amplification program was: pre-denaturation at 95 °C for 5 min, denaturation at 94 °C for 30 s, annealing at 55 °C for 30 s, extension at 72 °C for 1 min, with a total of 35 cycles; and a final extension at 72 °C for 10 min. After detection by 1% agarose gel electrophoresis, PCR products showing a single clear band of the expected size without non-specific amplification or primer-dimer formation were selected and sent to Sangon Biotech (Shanghai) Co., Ltd. for sequencing.

### 2.4. Data Processing

We sequenced 105 samples from 14 geographic localities to obtain *Cytb* sequences of *R. kukunoris*. After manual inspection of the sequencing chromatograms, the forward and reverse sequences were assembled and corrected using DNAMAN 8.0 software [26]. This 105-sequence dataset (final alignment length: 1144 bp) was used to calculate genetic diversity parameters, construct haplotype networks (using Popart-1.7), and perform Maximum Likelihood (ML) phylogenetic analysis. Furthermore, to more comprehensively evaluate the phylogenetic relationships, 160 mitochondrial *Cytb* sequences of *R. kukunoris* were retrieved from the NCBI GenBank database. These were combined with our 105 newly generated sequences to form a comprehensive dataset of 265 sequences. This combined dataset was aligned using MEGA11 [27] (final alignment length: 1141 bp) and was subsequently utilized for Maximum Parsimony (MP) phylogenetic analysis. DnaSP 6.0 [28] was used to calculate the number of variable sites (S), haplotype diversity (Hd), nucleotide diversity (Pi), average number of nucleotide differences (k), number of haplotypes (H), and nucleotide mismatch distribution. Arlequin 3.5 software [29] was used to calculate the distribution of molecular genetic variation among and within populations of *R. kukunoris* through Analysis of Molecular Variance (AMOVA), and also to calculate the genetic differentiation (Fst) values among localities, gene flow (Nm) among populations, and neutrality tests. Popart-1.7 was used to construct haplotype networks. Maximum Parsimony (MP) analysis was conducted in MEGA11 using the Close-Neighbor-Interchange (CNI) search method. Node support was evaluated with 1000 bootstrap replicates. Gaps were treated as missing data. Maximum Likelihood (ML) analysis was also performed in MEGA11. The best-fit nucleotide substitution model was selected based on the Bayesian Information Criterion (BIC), and the Kimura 2-parameter (K2P) model was determined to be optimal. The ML tree was reconstructed under the K2P model with 1000 bootstrap replicates to assess branch support. The phylogenetic tree was visually edited using Figtree v1.4.4. Historical changes in effective population size (*N_e_*) were reconstructed using a coalescent-based approach implemented in MATLAB R2022b. The mutation rate was set to 1.0 × 10^−8^ substitutions per site per year, with a generation time of one year. Time was scaled in million years (Mya). Markov Chain Monte Carlo (MCMC) sampling (5000 iterations) and bootstrap resampling (*n* = 300) were performed separately for each population to estimate *N_e_* trajectories and their confidence intervals.

## 3. Results

### 3.1. PCR Amplification and Sequence Variation Analysis

The amplified mitochondrial *Cytb* gene fragment of *R. kukunoris* was approximately 1100 bp in length. After sequence assembly and manual correction, 105 complete *Cytb* sequences were obtained, each with a final length of 1144 bp. Base composition analysis showed that A, T, C, and G accounted for 24.75%, 30.03%, 31.57%, and 13.65%, respectively; the overall A + T content was 54.78%, and the GC content was 45.22%, indicating an A + T bias (Table 2).

A total of 26 variable sites were identified among the 105 *Cytb* sequences. Of these, 16 sites (301, 441, 469, 512, 632, 633, 638, 698, 772, 778, 832, 847, 863, 879, 1020, and 1035) were single-nucleotide polymorphic sites with two nucleotide states. Additionally, nine parsimony-informative sites with two nucleotide states were detected (160, 325, 370, 472, 528, 709, 710, 998, and 1027), and one parsimony-informative site with three nucleotide states was identified (868). No sites with four nucleotide states were observed (Table 3). The mitochondrial *Cytb* sequences generated in this study have been deposited in the Genome Sequence Archive (GSA) at the National Genomics Data Center (NGDC), China National Center for Bioinformation, under accession numbers CRX2464014-CRX2466400.

### 3.2. Analysis of Genetic Diversity

Genetic parameters of 14 geographic *R. kukunoris* populations were calculated using DnaSP 6.0 software. Sequence analysis identified a total of 21 haplotypes. For the mitochondrial *Cytb* gene across the 14 populations, the total haplotype diversity (h) was 0.735, nucleotide diversity (π) was 0.00166, and the average number of nucleotide differences (k) was 1.899.

Among these 14 populations, the Tongren population exhibited the highest haplotype diversity, while the Gande population showed the lowest. The Jiuzhi-Baiyu population had the highest nucleotide diversity, and the Zeku population had the lowest (Table 4).

### 3.3. Analysis of Population Structure

AMOVA was performed using Arlequin 3.5 with sampling populations treated as populations, without defining higher-level groups. The results showed that, of the total genetic variation in the mitochondrial *Cytb* gene, variation among populations accounted for 31.32%, whereas variation within populations accounted for 68.68%. This indicates that genetic diversity in *R. kukunoris* is primarily distributed within populations, with relatively low differentiation among populations (Table 5). Pairwise Fst values ranged from negative values to 1. Negative Fst values (e.g., HN vs. ZK = −0.03922) were interpreted as indicating no genetic differentiation, as such values are generally attributed to sampling variance rather than true population divergence. Regarding pairwise population differentiation, the Fst value between the Wulan and Maqin populations was the highest (Fst = 1, *p* < 0.05) with a gene flow value (Nm) of 0. Additionally, the Wulan and Zeku populations exhibited an Fst of 0.92875 (*p* < 0.05) and Nm of 0.01918, while the Gonghe and Wulan populations showed an Fst of 0.92308 (*p* < 0.05) and Nm of 0.02083. AMOVA analysis revealed the highest genetic differentiation coefficient between the Wulan and Maqin populations, indicating significant genetic divergence between them. Furthermore, Nm < 1 suggests that there is virtually no gene flow among these populations (Table 6).

### 3.4. Inference of Historical Population Dynamics Based on Neutrality Tests and Mismatch Distribution

Analysis of the historical population dynamics of 14 *R. kukunoris* populations using Arlequin 3.5 software yielded the following results: Tajima’s D and Fu’s Fs are sensitive indicators of historical demographic events. Negative values generally suggest population expansion or purifying selection, whereas positive values may reflect population contraction, balancing selection, or population structure. Neutrality tests (Table 7) showed that Tajima’s D values ranged from −1.51227 to 1.81122, with no statistical significance observed across all populations. Fu′s Fs values ranged from −1.54636 to 1.60944, and the Fu′s Fs test for the Zeku population was statistically significant, indicating that the genetic structure of this population has been significantly affected by historical demographic dynamics. In the present study, most Tajima’s D and Fu’s Fs values were negative but not statistically significant, indicating that the populations of *R. kukunoris* may have experienced recent demographic expansion, although the signal is relatively weak.

**Figure 2:**
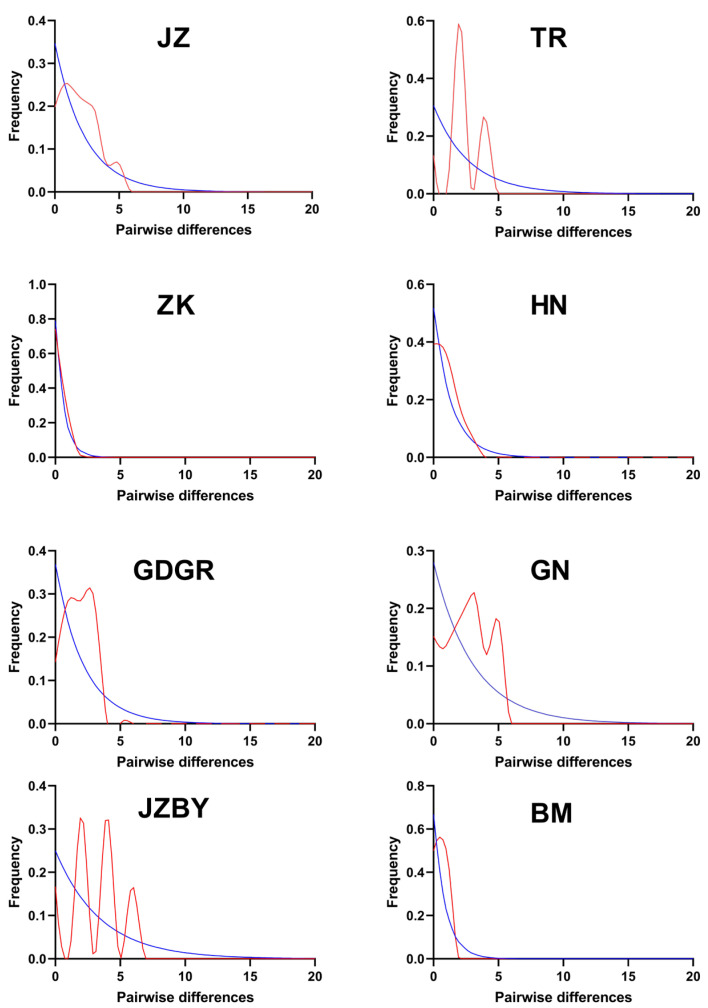

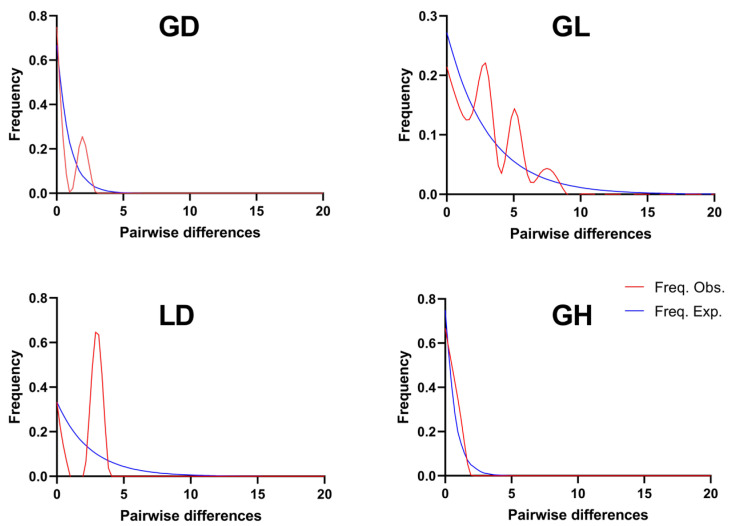
Distribution of nucleotide mismatch in population of *R. kukunoris*.

The mismatch distribution analysis based on the *Cytb* gene for 14 populations of *R. kukunoris* (Figure 2) revealed that some populations exhibited a multimodal pattern. The overall mismatch distribution patterns of the JZ, TR, GDGR, GN, JZBY, GD, GL, and LD populations exhibited a multimodal trend. These results suggest that *R. kukunoris* populations may have experienced historical population expansion events.

**Figure 3:**
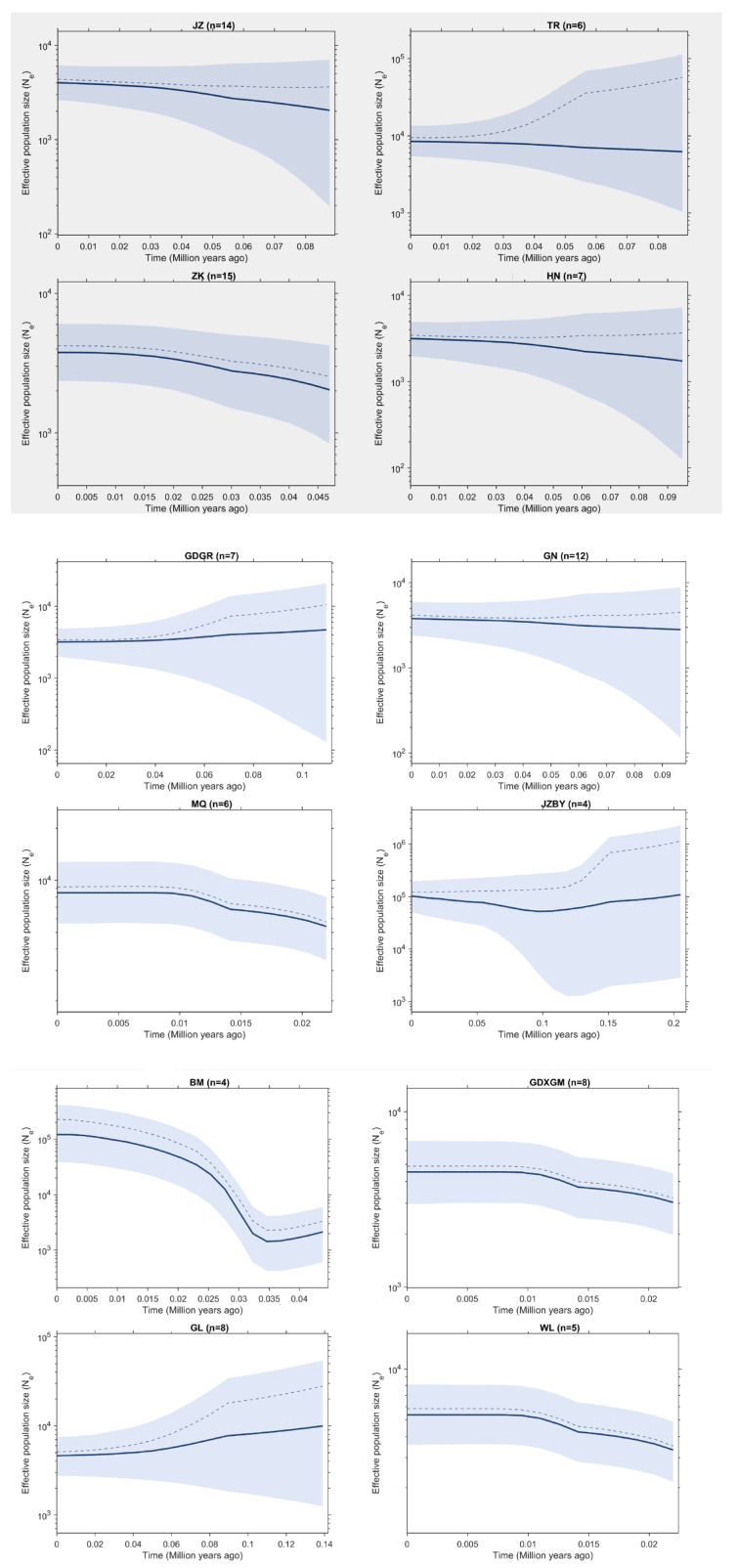

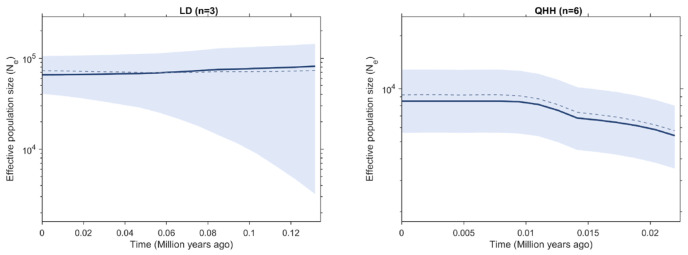
Historical changes in effective population size (*N_e_*) of *R. kukunoris* populations. **Note:** Dashed line indicates the midpoint of the interval; solid line shows the median.

To further investigate historical demographic trends, changes in effective population size (*N_e_*) through time were reconstructed based on mitochondrial *Cytb* sequences (Figure 3) [30]. Most populations exhibited relatively stable or gradually declining *N_e_* toward the present, and no strong, synchronous expansion signal was detected. This pattern is consistent with the non-significant neutrality tests and the multimodal mismatch distributions observed above. Several populations, including JZ, ZK, HN, GN, MQ, QHH, WL, and GDXGM, showed decreasing *N_e_* toward the present. The BM population exhibited the most pronounced fluctuation, with a marked reduction in *N_e_* around 0.03–0.04 Mya, followed by a slight recovery. In contrast, GL and GDGR showed relatively stable to slightly increasing *N_e_* through time. Populations of the northern lineage (e.g., from the northeastern Qinghai-Xizang Plateau and the Hexi Corridor) exhibited stable or slightly declining effective population sizes (*N_e_*), suggesting that these regions likely served as glacial refugia with relatively minor demographic fluctuations. In contrast, populations of the southern lineage showed more pronounced fluctuations in *N_e_*, which may be associated with postglacial climatic warming and population expansion in these areas.

Based on the Kimura two-parameter (K2P) substitution model, genetic distances among *R. kukunoris* populations were calculated using MEGA 11.0 (Table 8). Genetic distances ranged from 0.0001 to 0.0038, with the largest value (0.0038) observed between the Guinan and Wulan populations and the smallest (0.0001) between the Zeku and Maqin populations.

The evolutionary rate was inferred from the reported range of 2–4.4% per million years (Myr) for the mitochondrial *Cytb* gene in frogs [31]. Divergence times were estimated using the formula t = D/2a, where t represents divergence time, D denotes genetic distance, and a corresponds to the molecular clock rate. This approach was applied to infer the divergence times among geographic populations of *R. kukunoris*.

The estimated divergence time between the Guinan and Wulan populations was approximately 0.043–0.095 million years ago (Mya), reflecting their relatively large genetic distance. In contrast, the Zeku and Maqin populations showed the smallest divergence time, estimated at approximately 0.0011–0.0025 Mya.

### 3.5. Phylogenetic Analysis

**Figure 4:**
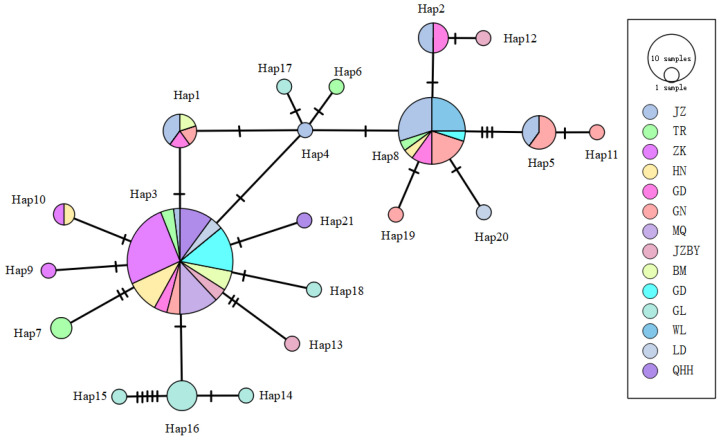
The haplotype network of *Cytb* gene sequences of *R. kukunoris*.

Analysis of the haplotype network for the 21 haplotypes identified across 14 *R. kukunoris* populations (Figure 4) revealed a radiating pattern centered on Hap3 and Hap8, with a relatively dispersed network structure. This pattern suggests that these haplotypes may represent ancestral haplotypes of *R. kukunoris.*

**Figure 5:**
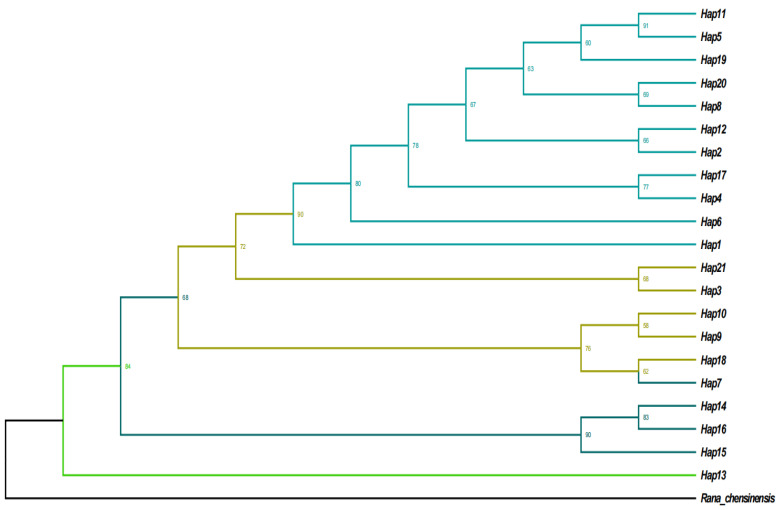
ML phylogenetic tree of *R. kukunoris* haplotypes based on mtDNA *Cytb* sequences.

Using *R. chensinensis* as the outgroup, a maximum likelihood (ML) phylogenetic tree was constructed based on the 21 *Cytb* haplotypes of *R. kukunoris*. The results (Figure 5) showed that the haplotypes were grouped into four distinct clades. H1, H11, H5, H19, H20, H8, H12, H2, H17, H4, and H6 clustered together, corresponding to populations from Jianzha, Guide, Guinan, Banma, Wulan, Ledu, and Gande. H21, H3, H10, H9, and H18 formed a second clade, including populations from Maqin, Zeku, Henan, and Gonghe. H7, H14, H16, and H15 constituted a third clade corresponding to the Tongren and Gulang populations, whereas H13 formed a distinct single-member clade representing the Jiuzhi-Baiyu population.

A total of 160 mitochondrial DNA *Cytb* sequences of *R. kukunoris* were retrieved from the NCBI database (Table S1). Combined with the 105 sequences amplified in the present study, a total of 265 *Cytb* sequences were subjected to haplotype analysis using DnaSP 6.0 software, which identified 57 distinct haplotypes. A phylogenetic tree was constructed based on these *Cytb* gene sequences using MEGA11 with the maximum parsimony (MP) method. Phylogenetic analysis revealed that the 265 *Cytb* sequences clustered into two distinct clades.

**Figure 6:**
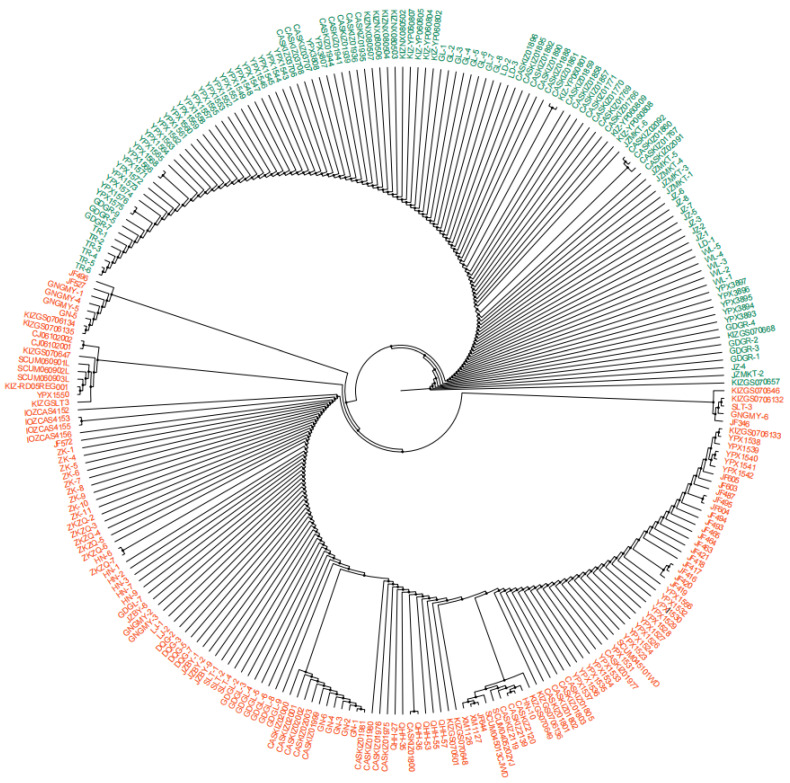
MP tree of *R. kukunoris* based on sequences of mtDNA *Cytb*. **Note:** Two different colors represent different branches; dark green represents branch A, and orange-red represents branch B. Two different colors represent different branches; dark green represents Clade A, and orange-red represents Clade B. Bootstrap values for major nodes are indicated by symbols (● for 70–90%).

Based on sampling locations, Clade A included populations from: Dingxi Longxi, Dingxi Daping, Tianshui Qinzhou, Linxia Yongjing, Datong, Huangzhong Jingfang Village, Haibei Prefecture Xihai Town, Haibei Prefecture Bird Island, Haidong Huzhu, Haidong Ledu, Yinchuan Gunzhongkou, Yongdeng, Wuwei Tianzhu County, Gulang, Wulan, Jianzha, Tongren, and Guide Garang. In contrast, Clade B comprised populations from: Guanmotan; Sangke, Xiahe, Lintan, and Zhaoni County (Gannan Prefecture); Toletai, Ziketan, and Gonghe (Hainan Prefecture); Litang (Ganzi Prefecture); Maowen and Wanglang (Mianyang); Hongyuan County, Ruoergai, 70 km northwest of Ruoergai, Miyaro, Songgang, Ako’er, Mengbishan, Huanglongshan, and Gonggaling (Aba Prefecture); Baimagou (Longnan); Guinan and Guinan Guomaying; Zeku Heri Town and Zequ Town (Zeku County); Huangnan Henan; and Guoluo Maqin Lajia Town, Guoluo Maqin Dongqing Gully, Guoluo Jiuzhi Baiyu, Guoluo Banma Sailaitang, and Guoluo Gande Ganglong (Figure 6).

Clade A represents the northern lineage, whereas Clade B represents the southern lineage. The two lineages are separated by an elevational boundary at approximately 3200 m. This pattern may be associated with postglacial processes in plateau forest frogs. After the end of the glacial period, part of the population likely migrated southward and adapted to local environments in regions such as southern Qinghai, southern Gansu, and northern Sichuan, leading to the formation of Clade B. Meanwhile, another portion of the population remained centered around glacial refugia in the Qilian Mountains and was distributed across low-elevation areas in the northeastern Qinghai-Xizang Plateau and riverine oases of the Hexi Corridor, forming Clade A.

## 4. Discussion

### 4.1. Analysis of Genetic Diversity and Genetic Structure of R. kukunoris Populations

In genetic diversity analyses, haplotype diversity and nucleotide diversity are commonly used indices to evaluate genetic variation within sampling populations. Following PCR amplification, sequencing, and alignment, 105 complete *Cytb* gene sequences were obtained from *R. kukunoris*. It should be noted that the genetic diversity and population structure analyses were conducted exclusively on these 105 newly sequenced individuals. This is because our samples represent standardized, systematic population-level sampling across 14 distinct populations. Incorporating the additional 160 sequences retrieved from GenBank—which are often opportunistic and exhibit uneven sample sizes across varying geographic scales—would introduce severe sampling bias and artificially skew the population genetic parameters. Across the 14 sampled populations, 26 variable sites and 21 haplotypes were identified, yielding an overall haplotype diversity (Hd) of 0.735 and nucleotide diversity (π) of 0.00166. This pattern suggests that these populations may have experienced a historical bottleneck followed by rapid demographic expansion [32,33].

Genetic distance reflects the degree of relatedness among groups [34], and the genetic differentiation coefficient (FST) serves as an important indicator of population divergence. Based on AMOVA results and significant differentiation among groups, the sampled populations can be regarded as genetically structured populations. Variation within populations (68.68%) exceeded that among populations (31.32%), indicating that most genetic diversity is maintained within populations. The estimated gene flow (Nm = 0.55) further suggests limited genetic exchange among populations [35]. Notably, the Wulan population inhabits the eastern margin of the Qaidam Basin, an arid and saline-alkali region representing the westernmost distributional limit of the species on the Qinghai-Xizang Plateau. Gene flow between the Wulan population and other conspecific populations is highly restricted, particularly between Wulan and Maqin (FST = 1, Nm = 0), indicating an almost complete absence of genetic exchange between these two lineages.

Zhou et al. proposed that uplift of the Qinghai-Xizang Plateau may have driven population differentiation in *R. kukunoris* [12]. Similarly, Ye et al. suggested that geographic barriers likely play a central role in shaping genetic structure in amphibian populations [36]. Wu et al. attributed genetic differentiation in the marsh tree frog to geographic isolation on Mount Fanjing, where most variation occurred among rather than within populations [37]. Taken together, these findings suggest that the high elevations and deeply incised valleys of the Qinghai-Xizang Plateau form pronounced geographic barriers. Given the generally limited dispersal capacity of plateau amphibians, such landscape features likely restrict gene flow and promote genetic differentiation among populations.

### 4.2. Population Historical Dynamics

Neutrality test results showed that Tajima’s D and Fu’s Fs values were not statistically significant for most populations. Only the Zeku population exhibited a significant Fu’s Fs value (*p* < 0.05), suggesting potential historical demographic influences, such as population expansion or bottlenecks, as well as possible signals of non-neutral evolution. In the northeastern Xizang Plateau, species population dynamics are often associated with Quaternary glacial refugia and postglacial expansion processes [38]. Studies on the yellow-bristled pika in northeastern Xizang have similarly demonstrated that regional environmental changes directly drive population expansion or contraction [39]. Likewise, research on plateau pikas indicates that topographic barriers resulting from uplift of the northeastern Xizang Plateau, together with Quaternary climatic changes, jointly shaped their postglacial expansion. During this process, five distinct lineages emerged, promoting regional population differentiation and phased expansion events [40]. These findings further suggest that the primary drivers of population differentiation and genetic structure in species of the northern Xizang Plateau are the combined effects of tectonic activity and Quaternary climatic fluctuations [41].

In this study, the nucleotide mismatch distribution exhibited a multimodal pattern. Analysis of *Cytb* sequence variation suggests that *R. kukunoris* may have experienced one or more population expansion events, likely associated with Quaternary climatic variability and glacial retreat on the Xizang Plateau. This interpretation is consistent with the proposed model in which “barriers such as the Qilian Mountains and the mainstem of the Yellow River, together with glacial climate,” shape population dynamics [42]. The reconstructed effective population size (*N_e_*) trajectories suggest that most populations of *R. kukunoris* did not experience a pronounced and synchronous demographic expansion. Rather, their demographic histories show evident regional differentiation, likely reflecting localized responses to climatic oscillations and the complex topographic barriers of the northeastern Qinghai-Xizang Plateau. Based on the reconstructed *N_e_* trajectories, gene flow between the northern and southern lineages appears to be strongly restricted, particularly across the elevational boundary at approximately 3200 m. This boundary, shaped by pronounced topographic barriers and hydrological fragmentation, likely plays a pivotal role in limiting genetic exchange between the two lineages. Consequently, populations within the northern lineage exhibit relatively stable demographic trends, whereas those in the southern lineage show greater historical fluctuations and signatures of localized expansion.

### 4.3. Phylogenetic Analysis of R. kukunoris

The eastern and southeastern margins of the Qinghai-Xizang Plateau, situated along its periphery, represent an important region for investigating mechanisms of speciation and population differentiation [43,44]. During glacial periods, some species persisted, leading to the formation of multiple refugia across the Qinghai-Xizang Plateau [45]. Refugia are areas that remained relatively stable throughout repeated glacial cycles and typically maintain high levels of genetic diversity. However, reductions in genetic diversity may occur when regional populations experience repeated postglacial expansions accompanied by bottleneck events [46,47].

Previous studies have shown that the northeastern Qinghai-Xizang Plateau functioned as a refugium for *R. kukunoris*. During the Last Glacial Period, the species likely persisted in the Qilian Mountains of the northeastern plateau. As glacial conditions ameliorated, populations dispersed and diverged into two distinct lineages: one subset migrated southward and differentiated into the southern (S) lineage, whereas the other remained in the Qilian Mountains and formed the northern (N) lineage [48]. The N lineage subsequently expanded northward along river systems originating in the Qilian Mountains into the Hexi Corridor, where multiple populations became established within riverine “islands” across the region.

The results of the present study support the division of *R. kukunoris* into northern and southern lineages; however, they differ from previous interpretations regarding the assignment of specific geographic populations. Zhou et al. suggested that the northern lineage comprised only populations distributed within the Hexi Corridor and that only two such populations existed, which were widely separated and strongly isolated, leading to the formation of internal sublineages [48]. In contrast, the present study included the Gulang population in Gansu Province, also located within the Hexi Corridor, and found that it clusters with populations from northeastern Qinghai Province and Yinchuan, together forming the northern lineage. populations from southern Qinghai Province, by comparison, cluster with those from northwestern Sichuan Province and the Hengduan Mountains, constituting the southern lineage. These two lineages are geographically separated by an elevational boundary at approximately 3200 m [49].

This biogeographic boundary, shaped by topographic barriers and hydrological fragmentation, substantially restricts gene flow between the two major lineages (Nm = 0.55) and appears to represent the primary geographic driver of lineage divergence. Although genetic differentiation between the two lineages is pronounced (FST = 0.31324), this boundary does not function as an absolute barrier. Moderate gene flow persists between the Tongren and Henan populations, as well as between the Guide and Guinan populations (Nm > 1), suggesting the presence of a narrow transitional zone where locally continuous habitats may facilitate limited genetic introgression between lineages. The establishment of this geographic boundary is likely the result of long-term synergistic interactions between tectonic movements and exogenic geomorphic processes. The demographic patterns inferred from *N_e_* trajectories further support this interpretation, as the two lineages display distinct historical population trends across the elevational divide. Furthermore, the multimodal pattern observed in the nucleotide mismatch distribution, together with the significant Fu’s Fs value for the Zeku population (−1.54636, *p* < 0.05), provides additional evidence that the interaction between population expansion processes and geographic barriers jointly drove the divergence of the two major genetic lineages. Although multilocus and genomic approaches are increasingly applied in phylogeographic research, mitochondrial *Cytb* remains a widely used marker due to its relatively high mutation rate and effectiveness in detecting historical lineage divergence. However, we acknowledge that reliance on a single mitochondrial gene reflects only maternal inheritance patterns. Future studies integrating nuclear markers or genome-wide data would further improve resolution and provide a more comprehensive view of population history. Collectively, these findings offer important empirical support for understanding the evolutionary origin of *R. kukunoris* and for informing future conservation efforts.

## 5. Conclusions

Based on mitochondrial *Cytb* sequences from 14 populations in the northeastern Qinghai-Xizang Plateau, this study reveals a clear phylogeographic structure in *R. kukunoris*. The species exhibits moderate haplotype diversity and low nucleotide diversity, consistent with historical demographic contraction followed by limited expansion. Population genetic analyses indicate moderate genetic differentiation and restricted gene flow, with particularly strong isolation in certain geographically marginal populations. Phylogenetic analyses support the division of *R. kukunoris* into northern and southern lineages separated by an elevational boundary at approximately 3200 m, which functions as an important geographic barrier to gene exchange. Historical demographic reconstructions further reveal regionally distinct population trajectories rather than a unified expansion pattern. Overall, the interaction between plateau uplift, complex topography, and Quaternary climatic oscillations appears to have shaped the present genetic structure of this species. These results provide a robust genetic framework for future evolutionary and conservation studies of *R. kukunoris*.

## Figures and Tables

**Table 1:** The sample information.

Species Name	Sample Number	Locality Name	Altitude	Longitude/Latitude
*R. kukunoris*	GN-1, GN-2, GN-3, GN-4, GN-5, GN-6, GNGMY-1, GNGMY-2, GNGMY-3, GNGMY-4, GNGMY-5, GNGMY-6	Qinghai, Guinan	3500.00	E 101.2350 N 35.3624
*R. kukunoris*	LD-1, LD-2, LD-3	Qinghai, Ledu	2068.49	E 102.399387 N 36.542294
*R. kukunoris*	GL-1, GL-2, GL-3, GL-4, GL-5, GL-6, GL-7, GL-8	Gansu, Gulang	2260.86	E 103.002595 N 37.387218
*R. kukunoris*	JZ-1, JZ-2, JZ-3, JZ-4, JZ-5, JZ-6, JZ-7, JZ-8, JZMKT-1, JZMKT-2, JZMKT-3, JZMKT-4, JZMKT-5, JZMKT-6	Qinghai, Jianzha	1964.78	E 102.0222 N 35.5648
*R. kukunoris*	WL-1, WL-2, WL-3, WL-4, WL-5	Qinghai, Wulan	2906.40	E 98.490192 N 36.901527
*R. kukunoris*	GH-27, GH-35, GH-36, GH-53, GH-55, GH-57	South Shore of Qinghai Lake, Gonghe	3201.00	E 100.1224444 N 36.62405556
*R. kukunoris*	TR-1, TR-2, TR-3, TR-4, TR-5, TR-6	Qinghai, Tongren	2286.01	E 102.063597 N 35.630590
*R. kukunoris*	ZK-1, ZK-11, ZKZQ-2, ZKZQ-3, ZKZQ-4, ZKZQ-5, ZKZQ-6, ZKZQ-7	Qinghai, Zeku	3456.48	E 100.731466 N 35.15216
*R. kukunoris*	HN1-3, HN-6, HN-7, HN-9, HN-10	Qinghai, Henan	3471.46	E 101.545221 N 34.724452
*R. kukunoris*	GDGR1-5, GDGR-7, GDGR-9	Qinghai, Guide Garan	2644.70	E 101.614526 N 36.238186
*R. kukunoris*	LJ-1, LJ-2, DQJ-2, DQJ-3, DQJ-5, DQJ-7	Qinghai, Maqin	4038.35	E 100.675290 N 34.692199
*R. kukunoris*	JZBY-1, JZBY-2, JZBY-6, JZBY-9	Qinghai, Jiuzhi Baiyu	4228.96	E 100.5241428 N 33.175289
*R. kukunoris*	SLT-1, SLT-2, SLT-3, SLT-4	Qinghai, Banma	3540.99	E 100.733374 N 32.949384
*R. kukunoris*	GD-2, GD-3, GD-4, GD-5, GD-6, GD-7, GD-8, GD-9	Qinghai, Gande	3999.91	E 100.43913 N 33.532893

**Note:** The “labels” represent the localities assigned to each sampling locality, while the adjacent numbers correspond to the individual sample identifiers.

**Table 2:** Base composition of *Cytb* sequences in fourteen populations of *R. kukunoris*.

Populations	A/%	T/%	C/%	G/%	A + T/%
JZ	24.77	29.98	31.61	13.62	54.75
TR	24.76	29.99	31.62	13.63	54.75
ZK	24.72	30.06	31.56	13.67	54.78
HN	24.73	30.04	31.58	13.66	54.76
GDGR	24.73	29.98	31.54	13.68	54.71
GN	24.81	29.99	31.60	13.60	54.80
MQ	24.72	30.06	31.55	13.67	54.78
JZBY	24.74	30.04	31.53	13.69	54.78
BM	24.72	30.04	31.55	13.69	54.75
GD	24.73	30.05	31.56	13.66	54.78
GL	24.74	30.09	31.50	13.67	54.83
WL	24.80	29.97	31.64	13.58	54.78
LD	24.77	30.03	31.55	13.64	54.81
GH	24.72	30.08	31.54	13.67	54.79
Average	24.75	30.03	31.57	13.65	54.78

**Table 3:** Summary of variable sites detected in 105 *Cytb* sequences of *R. kukunoris*.

Site Category	Position(s)	No. of Sites	No. of Nucleotide States
Variable sites (2 states, non-parsimony-informative)	301, 441, 469, 512, 632, 633, 638, 698, 772, 778, 832, 847, 863, 879, 1020, 1035	16	2
Parsimony-informative sites (2 states)	160, 325, 370, 472, 528, 709, 710, 998, 1027	9	2
Parsimony-informative site (3 states)	868	1	3
Total variable sites	—	26	—

**Table 4:** Measures of mitochondrial DNA *Cytb* genetic diversity observed in *R. kukunoris*.

Populations	Number of Samples	Number of Haplotypes	Haplotype	Haplotype Diversity (h)	Nucleotide Diversity (π)
JZ	14	6	Hap1, Hap2, Hap3, Hap4, Hap5, Hap8	0.802	0.00165
TR	6	4	Hap3, Hap6, Hap7, Hap8	0.867	0.00199
ZK	15	3	Hap3, Hap9, Hap10	0.275	0.00023
HN	7	3	Hap3, Hap8, Hap10	0.524	0.00075
GDGR	7	4	Hap1, Hap2, Hap3, Hap8	0.857	0.00150
GN	12	6	Hap1, Hap3, Hap5, Hap8, Hap11, Hap19	0.848	0.00226
MQ	6	1	Hap3	-	-
JZBY	4	3	Hap3, Hap12, Hap13	0.833	0.00263
BM	4	2	Hap1, Hap3	0.500	0.00044
GD	8	2	Hap3, Hap8	0.250	0.00044
GL	8	5	Hap14, Hap15, Hap16, Hap17, Hap18	0.786	0.00235
WL	5	1	Hap8	-	-
LD	3	2	Hap3, Hap20	0.667	0.00175
GH	6	5	Hap3, Hap21	0.333	0.00029
Total	105	21	21	0.735	0.00166

**Table 5:** AMOVA analysis among populations and within populations in *R. kukunoris*.

Source of Variation	d.f.	Sum of Squares	Variance Components	Percentage of Variation
Among populations	13	37.906	0.30493 Va	31.32
Within populations	91	60.837	0.66854 Vb	68.68
Total	104	98.743	0.97346	100
Fixation Index			FST: 0.31324	

**Table 6:** Fst values among populations in *R. kukunoris*.

	JZ	TR	ZK	HN	GDGR	GN	MQ	JZBY	BM	GD	GL	WL	LD	GH
JZ		+	+	+	-	-	+	+	+	+	+	+	+	+
TR	0.24294		+	+	+	+	+	-	+	+	+	+	-	+
ZK	0.53828	0.13448		-	+	+	-	-	-	-	+	+	-	-
HN	0.37639	0.03529	−0.03922		+	+	-	-	-	-	+	+	-	-
GDGR	−0.02370	0.12917	0.41401	0.22222		+	+	-	+	+	+	+	-	+
GN	−0.01138	0.25501	0.48884	0.35633	0.09909		+	+	+	+	+	+	+	+
MQ	0.57568	0.15000	0.00000	0.00000	0.45455	0.51705		-	-	-	+	+	-	-
JZBY	0.14615	−0.01935	0.00000	−0.08000	−0.01538	0.18404	0.00000		-	-	+	+	-	-
BM	0.45841	0.07778	0.00000	−0.05556	0.31111	0.42330	0.00000	−0.03704		-	+	+	-	-
GD	0.42573	0.05143	0.00000	−0.11765	0.27059	0.39493	0.00000	−0.07692	−0.06667		+	+	-	-
GL	0.41191	0.20876	0.26405	0.22047	0.33152	0.39663	0.28571	0.14286	0.24094	0.24094		+	+	+
WL	0.12308	0.51429	0.92857	0.76923	0.25000	0.22727	1.00000	0.40000	0.87500	0.85714	0.63054		+	+
LD	0.10482	−0.06667	0.00000	−0.15385	−0.05405	0.14205	0.00000	−0.15385	−0.07143	−0.15385	0.16205	0.40000		-
GH	0.53538	0.13333	0.00000	0.00000	0.41096	0.48663	0.00000	0.00000	0.00000	0.00000	0.26239	0.92308	0.00000	

**Note:** Data below the diagonal represent pairwise Fst values, while data above the diagonal indicate Fisher’s exact test results. “+” indicates a significant difference (*p* < 0.05), and “-” indicates no significant difference (*p* > 0.05).

**Table 7:** Neutrality tests of 14 populations.

	JZ	TR	ZK	HN	GDGR	GN	MQ	JZBY	BM	GD	GL	WL	LD	GH	Total Population
Tajima’s D	−0.01445	0.19651	−1.49051	−1.35841	1.81122	1.13044	0.00000	−0.80861	−0.61237	−1.31009	−1.51227	0.00000	0.00000	−0.93302	−0.35011
Fu’s Fs	−1.13612	−0.27174	−1.54636 *	−0.23726	−0.42812	−0.67865	0.00000	0.73089	0.17185	0.76178	−0.52342	0.00000	1.60944	−0.00275	−0.11075

**Note:** “*” indicates a significant difference (*p* < 0.05).

**Table 8:** Kimura’s two-parameter genetic distances of populations in *R. kukunoris* based on mtDNA *Cytb* sequences.

	JZ	TR	ZK	HN	GDGR	GN	MQ	JZBY	BM	GD	GL	WL	LD	GH
JZ		0.0009	0.0010	0.0009	0.0006	0.0008	0.0010	0.0009	0.0010	0.0009	0.0011	0.0004	0.0008	0.0010
TR	0.0024		0.0005	0.0006	0.0008	0.0010	0.0005	0.0007	0.0006	0.0006	0.0009	0.0010	0.0007	0.0005
ZK	0.0021	0.0013		0.0002	0.0008	0.0011	0.0001	0.0005	0.0002	0.0002	0.0007	0.0012	0.0005	0.0002
HN	0.0019	0.0014	0.0005		0.0007	0.0010	0.0002	0.0006	0.0003	0.0003	0.0007	0.0010	0.0005	0.0003
GDGR	0.0015	0.0020	0.0015	0.0014		0.0008	0.0008	0.0008	0.0008	0.0007	0.0010	0.0005	0.0007	0.0008
GN	0.0019	0.0028	0.0025	0.0023	0.0021		0.0011	0.0010	0.0011	0.0010	0.0012	0.0006	0.0009	0.0011
MQ	0.0019	0.0012	0.0001	0.0004	0.0014	0.0023		0.0005	0.0002	0.0001	0.0007	0.0012	0.0005	0.0002
JZBY	0.0025	0.0023	0.0014	0.0016	0.0020	0.0030	0.0013		0.0006	0.0005	0.0008	0.0009	0.0007	0.0005
BM	0.0019	0.0013	0.0003	0.0006	0.0014	0.0023	0.0002	0.0015		0.0003	0.0007	0.0012	0.0006	0.0003
GD	0.0018	0.0013	0.0003	0.0005	0.0013	0.0022	0.0002	0.0014	0.0004		0.0007	0.0010	0.0005	0.0002
GL	0.0034	0.0027	0.0018	0.0020	0.0029	0.0038	0.0016	0.0029	0.0018	0.0018		0.0013	0.0008	0.0007
WL	0.0009	0.0020	0.0019	0.0016	0.0010	0.0015	0.0018	0.0022	0.0018	0.0015	0.0032		0.0008	0.0012
LD	0.0019	0.0018	0.0010	0.0011	0.0015	0.0023	0.0009	0.0019	0.0010	0.0009	0.0024	0.0015		0.0005
GH	0.0021	0.0013	0.0003	0.0005	0.0015	0.0025	0.0001	0.0015	0.0004	0.0004	0.0018	0.0019	0.0010	

## Data Availability

Data available in a publicly accessible repository. https://ngdc.cncb.ac.cn/gsub/ (accessed on 23 January 2026).

## Supplementary Materials

The following supporting information can be downloaded at: https://www.mdpi.com/article/10.3390/ani16071013/s1, Table S1: Information for *Rana kukunoris* included in this study.
