# Supplementary material for: Phylogeography and Genetic Diversity of Rana kukunoris on the Northeast Qinghai-Xizang Plateau: Insights from Mitochondrial Cytochrome b Gene
Source: Animals (Basel). 2026 Mar 26;16(7):1013. doi: 10.3390/ani16071013 (PMC13072228; doi:10.3390/ani16071013)
Supplement: Supplementary file 1 [file animals-16-01013-s001.zip › animals-4142028-supplementary.pdf]

TableS1 Information for *Rana kukunoris* included in this study.

Number	Speciesname	Locality	Samplenumber	Haplotype	Accession number	Database
1	*R.kukunoris*	Guanmotan, Lanzhou, Gansu, China	KIZGS070601	H3	JN984158	GenBank
2	*R.kukunoris*	Guanmotan, Lanzhou, Gansu, China	KIZGS070646	H8	JN984159	GenBank
3	*R.kukunoris*	Guanmotan, Lanzhou, Gansu, China	KIZGS070647	H20	JN984160	GenBank
4	*R.kukunoris*	Guanmotan, Lanzhou, Gansu, China	KIZGS070648	H3	JN984161	GenBank
5	*R.kukunoris*	Guanmotan, Lanzhou, Gansu, China	KIZGS070649	H21	JN984162	GenBank
6	*R.kukunoris*	Sangke, Xiahe, Gannan, Gansu, China	KIZGS0706132	H8	JN984163	GenBank
7	*R.kukunoris*	Sangke, Xiahe, Gannan, Gansu, China	KIZGS0706133	H22	JN984164	GenBank
8	*R.kukunoris*	Sangke, Xiahe, Gannan, Gansu, China	KIZGS0706134	H8	JN984165	GenBank
9	*R.kukunoris*	Sangke, Xiahe, Gannan, Gansu, China	KIZGS0706135	H23	JN984166	GenBank
10	*R.kukunoris*	Sangke, Xiahe, Gannan, Gansu, China	KIZGS0706136	H3	JN984167	GenBank
11	*R.kukunoris*	Longxi, Dingxi, Gansu, China	CASKIZ02091	H8	JN984168	GenBank
12	*R.kukunoris*	Longxi, Dingxi, Gansu, China	CASKIZ02092	H24	JN984169	GenBank
13	*R.kukunoris*	Qinzhou, Tianshui, Gansu, China	KIZGS070657	H25	JN984170	GenBank
14	*R.kukunoris*	Qinzhou, Tianshui, Gansu, China	KIZGS070668	H26	JN984171	GenBank
15	*R.kukunoris*	Qinzhou, Tianshui, Gansu, China	CASKIZ02119	H27	JN984172	GenBank

Number	Speciesname	Locality	Samplenumber	Haplotype	Accession number	Database
16	*R.kukunoris*	Qinzhou, Tianshui, Gansu, China	CASKIZ02120	H28	JN984173	GenBank
17	*R.kukunoris*	Qinzhou, Tianshui, Gansu, China	CASKIZ02139	H29	JN984174	GenBank
18	*R.kukunoris*	Lintan, Gannan, Gansu, China	KIZGSLT3	H30	JN984175	GenBank
19	*R.kukunoris*	Yongjing, Linxia, Gansu, China	KIZ-YP060808	H8	JN984176	GenBank
20	*R.kukunoris*	Yongjing, Linxia, Gansu, China	KIZ-YP060809	H8	JN984177	GenBank
21	*R.kukunoris*	Datong, Xining, Qinghai, China	CASKIZ01766	H8	JN984178	GenBank
22	*R.kukunoris*	Datong, Xining, Qinghai, China	CASKIZ01767	H24	JN984179	GenBank
23	*R.kukunoris*	Datong, Xining, Qinghai, China	CASKIZ01769	H8	JN984180	GenBank
24	*R.kukunoris*	Datong, Xining, Qinghai, China	CASKIZ01770	H8	JN984181	GenBank
25	*R.kukunoris*	Datong, Xining, Qinghai, China	CASKIZ01771	H8	JN984182	GenBank
26	*R.kukunoris*	Southern Qinghai Lake, Gonghe, Qinghai, China	CASKIZ01800	H19	JN984183	GenBank
27	*R.kukunoris*	Southern Qinghai Lake, Gonghe, Qinghai, China	CASKIZ01801	H31	JN984184	GenBank
28	*R.kukunoris*	Southern Qinghai Lake, Gonghe, Qinghai, China	CASKIZ01802	H3	JN984185	GenBank
29	*R.kukunoris*	Southern Qinghai Lake, Gonghe, Qinghai, China	CASKIZ01803	H3	JN984186	GenBank
30	*R.kukunoris*	Southern Qinghai Lake, Gonghe, Qinghai, China	CASKIZ01805	H3	JN984187	GenBank
31	*R.kukunoris*	Jingfang Village, Huangzhong, Qinghai, China	CASKIZ01857	H8	JN984188	GenBank
32	*R.kukunoris*	Jingfang Village, Huangzhong, Qinghai, China	CASKIZ01858	H8	JN984189	GenBank

Number	Speciesname	Locality	Samplenumber	Haplotype	Accession number	Database
33	*R.kukunoris*	Jingfang Village, Huangzhong, Qinghai, China	CASKIZ01859	H32	JN984190	GenBank
34	*R.kukunoris*	Jingfang Village, Huangzhong, Qinghai, China	CASKIZ01860	H33	JN984191	GenBank
35	*R.kukunoris*	Jingfang Village, Huangzhong, Qinghai, China	CASKIZ01861	H34	JN984192	GenBank
36	*R.kukunoris*	Xihai Town, Haibei, Qinghai, China	CASKIZ01888	H8	JN984193	GenBank
37	*R.kukunoris*	Xihai Town, Haibei, Qinghai, China	CASKIZ01890	H8	JN984194	GenBank
38	*R.kukunoris*	Xihai Town, Haibei, Qinghai, China	CASKIZ01892	H8	JN984195	GenBank
39	*R.kukunoris*	Xihai Town, Haibei, Qinghai, China	CASKIZ01895	H35	JN984196	GenBank
40	*R.kukunoris*	Xihai Town, Haibei, Qinghai, China	CASKIZ01896	H8	JN984197	GenBank
41	*R.kukunoris*	Bird Island, Haibei, Qinghai, China	CASKIZ01935	H3	JN984198	GenBank
42	*R.kukunoris*	Bird Island, Haibei, Qinghai, China	CASKIZ01936	H3	JN984199	GenBank
43	*R.kukunoris*	Bird Island, Haibei, Qinghai, China	CASKIZ01939	H3	JN984200	GenBank
44	*R.kukunoris*	Bird Island, Haibei, Qinghai, China	CASKIZ01941	H3	JN984201	GenBank
45	*R.kukunoris*	Bird Island, Haibei, Qinghai, China	CASKIZ01944	H3	JN984202	GenBank
46	*R.kukunoris*	Tuoletai, Hainan, Qinghai, China	CASKIZ01975	H8	JN984203	GenBank
47	*R.kukunoris*	Tuoletai, Hainan, Qinghai, China	CASKIZ01976	H8	JN984204	GenBank
48	*R.kukunoris*	Tuoletai, Hainan, Qinghai, China	CASKIZ01977	H36	JN984205	GenBank
49	*R.kukunoris*	Tuoletai, Hainan, Qinghai, China	CASKIZ01980	H37	JN984206	GenBank
50	*R.kukunoris*	Tuoletai, Hainan, Qinghai, China	CASKIZ01981	H8	JN984207	GenBank
51	*R.kukunoris*	Ziketan, Hainan, Qinghai, China	CASKIZ01999	H38	JN984208	GenBank

Number	Speciesname	Locality	Samplenumber	Haplotype	Accession number	Database
52	*R.kukunoris*	Ziketan, Hainan, Qinghai, China	CASKIZ02000	H8	JN984209	GenBank
53	*R.kukunoris*	Ziketan, Hainan, Qinghai, China	CASKIZ02001	H8	JN984210	GenBank
54	*R.kukunoris*	Ziketan, Hainan, Qinghai, China	CASKIZ02002	H8	JN984211	GenBank
55	*R.kukunoris*	Ziketan, Hainan, Qinghai, China	CASKIZ02003	H39	JN984212	GenBank
56	*R.kukunoris*	Ziketan, Hainan, Qinghai, China	CJ06102001	H8	JN984213	GenBank
57	*R.kukunoris*	Qinghai Lake, Gonghe, Qinghai, China	CJ06102002	H8	JN984214	GenBank
58	*R.kukunoris*	Huzhu, Haidong, Qinghai, China	KIZ-YP060801	H3	JN984215	GenBank
59	*R.kukunoris*	Huzhu, Haidong, Qinghai, China	KIZ-YP060802	H16	JN984216	GenBank
60	*R.kukunoris*	Huzhu, Haidong, Qinghai, China	KIZ-YP060804	H8	JN984217	GenBank
61	*R.kukunoris*	Huzhu, Haidong, Qinghai, China	KIZ-YP060805	H40	JN984218	GenBank
62	*R.kukunoris*	Huzhu, Haidong, Qinghai, China	KIZ-YP060807	H41	JN984219	GenBank
63	*R.kukunoris*	Gunzhongkou, Yinchuan, Ningxia, China	KIZNX080502	H42	JN984220	GenBank
64	*R.kukunoris*	Gunzhongkou, Yinchuan, Ningxia, China	KIZNX080503	H43	JN984221	GenBank
65	*R.kukunoris*	Gunzhongkou, Yinchuan, Ningxia, China	KIZNX080504	H43	JN984222	GenBank
66	*R.kukunoris*	Gunzhongkou, Yinchuan, Ningxia, China	KIZNX080506	H43	JN984223	GenBank
67	*R.kukunoris*	Gunzhongkou, Yinchuan, Ningxia, China	KIZNX080507	H43	JN984224	GenBank
68	*R.kukunoris*	Litang, Ganzi, Sichuan, China	SCUM060901L	H8	JN984225	GenBank
69	*R.kukunoris*	Litang, Ganzi, Sichuan, China	SCUM060902L	H8	JN984226	GenBank

Number	Speciesname	Locality	Samplenumber	Haplotype	Accession number	Database
70	*R.kukunoris*	Litang, Ganzi, Sichuan, China	SCUM060903L	H8	JN984227	GenBank
71	*R.kukunoris*	Maowen, Mianyang, Sichuan, China	SCUM04050202YJ	H44	JN984228	GenBank
72	*R.kukunoris*	Hongyuan, Aba, Sichuan, China	SCUM045013CJWD	H45	JN984229	GenBank
73	*R.kukunoris*	Ruoergai, Aba, Sichuan, China	KIZ-RD05REG001	H46	JN984230	GenBank
74	*R.kukunoris*	Ruoergai, Aba, Sichuan, China	SCUM045101WD	H3	JN984231	GenBank
75	*R.kukunoris*	Miyaluo, Aba, Sichuan, China	YPX1523	H3	JN984232	GenBank
76	*R.kukunoris*	Miyaluo, Aba, Sichuan, China	YPX1524	H3	JN984233	GenBank
77	*R.kukunoris*	Miyaluo, Aba, Sichuan, China	YPX1526	H3	JN984234	GenBank
78	*R.kukunoris*	Miyaluo, Aba, Sichuan, China	YPX1527	H3	JN984235	GenBank
79	*R.kukunoris*	Miyaluo, Aba, Sichuan, China	YPX1528	H3	JN984236	GenBank
80	*R.kukunoris*	Songgang, Aba, Sichuan, China	YPX1529	H3	JN984237	GenBank
81	*R.kukunoris*	Songgang, Aba, Sichuan, China	YPX1530	H47	JN984238	GenBank
82	*R.kukunoris*	Songgang, Aba, Sichuan, China	YPX1531	H8	JN984239	GenBank
83	*R.kukunoris*	Songgang, Aba, Sichuan, China	YPX1532	H3	JN984240	GenBank
84	*R.kukunoris*	Songgang, Aba, Sichuan, China	YPX1533	H8	JN984241	GenBank
85	*R.kukunoris*	Songgang, Aba, Sichuan, China	YPX1534	H8	JN984242	GenBank
86	*R.kukunoris*	Akoli, Aba, Sichuan, China	YPX1535	H8	JN984243	GenBank
87	*R.kukunoris*	Akoli, Aba, Sichuan, China	YPX1536	H8	JN984244	GenBank
88	*R.kukunoris*	Akoli, Aba, Sichuan, China	YPX1537	H8	JN984245	GenBank
89	*R.kukunoris*	Akoli, Aba, Sichuan, China	YPX1538	H8	JN984246	GenBank
90	*R.kukunoris*	Akoli, Aba, Sichuan, China	YPX1539	H8	JN984247	GenBank

Number	Speciesname	Locality	Samplenumber	Haplotype	Accession number	Database
91	*R.kukunoris*	Akoli, Aba, Sichuan, China	YPX1540	H8	JN984248	GenBank
92	*R.kukunoris*	Seka, Ganzi, Sichuan, China	YPX1541	H8	JN984249	GenBank
93	*R.kukunoris*	Seka, Ganzi, Sichuan, China	YPX1542	H8	JN984250	GenBank
94	*R.kukunoris*	Seka, Ganzi, Sichuan, China	YPX1543	H48	JN984251	GenBank
95	*R.kukunoris*	Seka, Ganzi, Sichuan, China	YPX1544	H8	JN984252	GenBank
96	*R.kukunoris*	Seka, Ganzi, Sichuan, China	YPX1545	H8	JN984253	GenBank
97	*R.kukunoris*	Seka, Ganzi, Sichuan, China	YPX1546	H8	JN984254	GenBank
98	*R.kukunoris*	Yade, Ganzi, Sichuan, China	YPX1547	H8	JN984255	GenBank
99	*R.kukunoris*	Yade, Ganzi, Sichuan, China	YPX1548	H8	JN984256	GenBank
100	*R.kukunoris*	Yade, Ganzi, Sichuan, China	YPX1549	H8	JN984257	GenBank
101	*R.kukunoris*	Yade, Ganzi, Sichuan, China	YPX1550	H34	JN984258	GenBank
102	*R.kukunoris*	Yade, Ganzi, Sichuan, China	YPX1551	H8	JN984259	GenBank
103	*R.kukunoris*	Yade, Ganzi, Sichuan, China	YPX1552	H49	JN984260	GenBank
104	*R.kukunoris*	Nanduo, Ganzi, Sichuan, China	YPX1553	H8	JN984261	GenBank
105	*R.kukunoris*	Nanduo, Ganzi, Sichuan, China	YPX1555	H8	JN984262	GenBank
106	*R.kukunoris*	Nanduo, Ganzi, Sichuan, China	YPX1556	H3	JN984263	GenBank
107	*R.kukunoris*	Nanduo, Ganzi, Sichuan, China	YPX1557	H8	JN984264	GenBank
108	*R.kukunoris*	Nanduo, Ganzi, Sichuan, China	YPX1558	H8	JN984265	GenBank
109	*R.kukunoris*	Xiala, Ganzi, Sichuan, China	YPX1559	H8	JN984266	GenBank
110	*R.kukunoris*	Xiala, Ganzi, Sichuan, China	YPX1560	H8	JN984267	GenBank
111	*R.kukunoris*	Xiala, Ganzi, Sichuan, China	YPX1561	H8	JN984268	GenBank

Number	Speciesname	Locality	Samplenumber	Haplotype	Accession number	Database
112	*R.kukunoris*	Xiala, Ganzi, Sichuan, China	YPX1562	H8	JN984269	GenBank
113	*R.kukunoris*	Xiala, Ganzi, Sichuan, China	YPX1563	H8	JN984270	GenBank
114	*R.kukunoris*	Xiala, Ganzi, Sichuan, China	YPX1564	H8	JN984271	GenBank
115	*R.kukunoris*	Honglong Township, Ganzi, Sichuan, China	YPX1565	H8	JN984272	GenBank
116	*R.kukunoris*	Honglong Township, Ganzi, Sichuan, China	YPX1566	H50	JN984273	GenBank
117	*R.kukunoris*	Honglong Township, Ganzi, Sichuan, China	YPX1568	H50	JN984274	GenBank
118	*R.kukunoris*	Xinduqiao, Ganzi, Sichuan, China	YPX1571	H8	JN984275	GenBank
119	*R.kukunoris*	Xinduqiao, Ganzi, Sichuan, China	YPX1572	H8	JN984276	GenBank
120	*R.kukunoris*	Xinduqiao, Ganzi, Sichuan, China	YPX1573	H8	JN984277	GenBank
121	*R.kukunoris*	Xinduqiao, Ganzi, Sichuan, China	YPX1574	H51	JN984278	GenBank
122	*R.kukunoris*	Xinduqiao, Ganzi, Sichuan, China	YPX1575	H52	JN984279	GenBank
123	*R.kukunoris*	Xinduqiao, Ganzi, Sichuan, China	YPX1576	H52	JN984280	GenBank
124	*R.kukunoris*	Xinduqiao, Ganzi, Sichuan, China	JF346	H8	JN984281	GenBank
125	*R.kukunoris*	Xinduqiao, Ganzi, Sichuan, China	IOZCAS4152	H8	JN984282	GenBank
126	*R.kukunoris*	Xinduqiao, Ganzi, Sichuan, China	IOZCAS4153	H8	JN984283	GenBank
127	*R.kukunoris*	Xinduqiao, Ganzi, Sichuan, China	IOZCAS4155	H8	JN984284	GenBank
128	*R.kukunoris*	Xinduqiao, Ganzi, Sichuan, China	IOZCAS4156	H8	JN984285	GenBank
129	*R.kukunoris*	Mengbi Mountain, Aba, Sichuan, China	JF416	H53	JN984286	GenBank
130	*R.kukunoris*	Mengbi Mountain, Aba, Sichuan, China	JF417	H3	JN984287	GenBank

Number	Speciesname	Locality	Samplenumber	Haplotype	Accession number	Database
131	*R.kukunoris*	Mengbi Mountain, Aba, Sichuan, China	JF418	H3	JN984288	GenBank
132	*R.kukunoris*	Mengbi Mountain, Aba, Sichuan, China	JF419	H53	JN984289	GenBank
133	*R.kukunoris*	Mengbi Mountain, Aba, Sichuan, China	JF420	H53	JN984290	GenBank
134	*R.kukunoris*	Mengbi Mountain, Aba, Sichuan, China	JF421	H3	JN984291	GenBank
135	*R.kukunoris*	Huanglong Mountain, Aba, Sichuan, China	JF463	H3	JN984292	GenBank
136	*R.kukunoris*	Huanglong Mountain, Aba, Sichuan, China	JF464	H3	JN984293	GenBank
137	*R.kukunoris*	Huanglong Mountain, Aba, Sichuan, China	JF465	H3	JN984294	GenBank
138	*R.kukunoris*	Huanglong Mountain, Aba, Sichuan, China	JF493	H3	JN984295	GenBank
139	*R.kukunoris*	Huanglong Mountain, Aba, Sichuan, China	JF494	H3	JN984296	GenBank
140	*R.kukunoris*	Huanglong Mountain, Aba, Sichuan, China	JF495	H54	JN984297	GenBank
141	*R.kukunoris*	Huanglong Mountain, Aba, Sichuan, China	JF496	H8	JN984298	GenBank
142	*R.kukunoris*	Gongga Ridge, Aba, Sichuan, China	JF487	H55	JN984299	GenBank
143	*R.kukunoris*	70 km NW of Ruoergai, Aba, Sichuan, China	JF527	H8	JN984300	GenBank
144	*R.kukunoris*	Zhuoni, Gannan, Gansu, China	JF572	H8	JN984301	GenBank
145	*R.kukunoris*	Daping, Dingxi, Gansu, China	JF603	H3	JN984302	GenBank
146	*R.kukunoris*	Daping, Dingxi, Gansu, China	JF604	H54	JN984303	GenBank
147	*R.kukunoris*	Daping, Dingxi, Gansu, China	JF605	H3	JN984304	GenBank

Number	Speciesname	Locality	Samplenumber	Haplotype	Accession number	Database
148	*R.kukunoris*	Baimagou, Longnan, Gansu, China	JF644	H56	JN984305	GenBank
149	*R.kukunoris*	Wanglang, Mianyang, Sichuan, China	XM1126	H56	JN984306	GenBank
150	*R.kukunoris*	Wanglang, Mianyang, Sichuan, China	XM1127	H56	JN984307	GenBank
151	*R.kukunoris*	Yongdeng, Lanzhou, Gansu, China	YPX3808	H57	JN984308	GenBank
152	*R.kukunoris*	Yongdeng, Lanzhou, Gansu, China	YPX3807	H57	JN984309	GenBank
153	*R.kukunoris*	Yongdeng, Lanzhou, Gansu, China	CASKIZ03706	H57	JN984310	GenBank
154	*R.kukunoris*	Yongdeng, Lanzhou, Gansu, China	CASKIZ03707	H3	JN984311	GenBank
155	*R.kukunoris*	Yongdeng, Lanzhou, Gansu, China	CASKIZ03708	H3	JN984312	GenBank
156	*R.kukunoris*	Tianzhu, Wuwei, Gansu, China	YPX3893	H15	JN984313	GenBank
157	*R.kukunoris*	Tianzhu, Wuwei, Gansu, China	YPX3894	H15	JN984314	GenBank
158	*R.kukunoris*	Tianzhu, Wuwei, Gansu, China	YPX3895	H15	JN984315	GenBank
159	*R.kukunoris*	Tianzhu, Wuwei, Gansu, China	YPX3896	H15	JN984316	GenBank
160	*R.kukunoris*	Tianzhu, Wuwei, Gansu, China	YPX3897	H15	JN984317	GenBank
161	*R.kukunoris*	Gulang, Gansu, China	GL-1	H13	CRX2466361	NGDC
162	*R.kukunoris*	Gulang, Gansu, China	GL-2	H14	CRX2466362	NGDC
163	*R.kukunoris*	Gulang, Gansu, China	GL-3	H15	CRX2466363	NGDC
164	*R.kukunoris*	Gulang, Gansu, China	GL-4	H15	CRX2466364	NGDC
165	*R.kukunoris*	Gulang, Gansu, China	GL-5	H16	CRX2466365	NGDC
166	*R.kukunoris*	Gulang, Gansu, China	GL-6	H1	CRX2466366	NGDC

Number	Speciesname	Locality	Samplenumber	Haplotype	Accession number	Database
167	*R.kukunoris*	Gulang, Gansu, China	GL-7	H15	CRX2466367	NGDC
168	*R.kukunoris*	Gulang, Gansu, China	GL-8	H15	CRX2466368	NGDC
169	*R.kukunoris*	Guinan, Qinghai, China	GN-1	H8	CRX2466369	NGDC
170	*R.kukunoris*	Guinan, Qinghai, China	GN-2	H8	CRX2466370	NGDC
171	*R.kukunoris*	Guinan, Qinghai, China	GN-3	H8	CRX2466371	NGDC
172	*R.kukunoris*	Guinan, Qinghai, China	GN-4	H8	CRX2466372	NGDC
173	*R.kukunoris*	Guinan, Qinghai, China	GN-5	H5	CRX2466373	NGDC
174	*R.kukunoris*	Guinan, Qinghai, China	GN-6	H17	CRX2466374	NGDC
175	*R.kukunoris*	Wulan, Qinghai, China	WL-1	H8	CRX2466375	NGDC
176	*R.kukunoris*	Wulan, Qinghai, China	WL-2	H8	CRX2466376	NGDC
177	*R.kukunoris*	Wulan, Qinghai, China	WL-3	H8	CRX2466377	NGDC
178	*R.kukunoris*	Wulan, Qinghai, China	WL-4	H8	CRX2466378	NGDC
179	*R.kukunoris*	Wulan, Qinghai, China	WL-5	H8	CRX2466379	NGDC
180	*R.kukunoris*	Ledu, Qinghai, China	LD-1	H18	CRX2466380	NGDC
181	*R.kukunoris*	Ledu, Qinghai, China	LD-2	H3	CRX2466381	NGDC
182	*R.kukunoris*	Ledu, Qinghai, China	LD-3	H3	CRX2466382	NGDC
183	*R.kukunoris*	Jianzha, Qinghai, China	JZ-1	H8	CRX2466383	NGDC
184	*R.kukunoris*	Jianzha, Qinghai, China	JZ-2	H8	CRX2466384	NGDC
185	*R.kukunoris*	Jianzha, Qinghai, China	JZ-3	H8	CRX2466385	NGDC
186	*R.kukunoris*	Jianzha, Qinghai, China	JZ-4	H2	CRX2466386	NGDC
187	*R.kukunoris*	Jianzha, Qinghai, China	JZ-5	H8	CRX2466387	NGDC
188	*R.kukunoris*	Jianzha, Qinghai, China	JZ-6	H5	CRX2466388	NGDC

Number	Speciesname	Locality	Samplenumber	Haplotype	Accession number	Database
189	*R.kukunoris*	Jianzha, Qinghai, China	JZ-7	H8	CRX2466389	NGDC
190	*R.kukunoris*	Jianzha, Qinghai, China	JZ-8	H8	CRX2466390	NGDC
191	*R.kukunoris*	Qinghai Lake, Qinghai, China	QHH-27	H3	CRX2466391	NGDC
192	*R.kukunoris*	Qinghai Lake, Qinghai, China	QHH-35	H3	CRX2466392	NGDC
193	*R.kukunoris*	Qinghai Lake, Qinghai, China	QHH-36	H19	CRX2466393	NGDC
194	*R.kukunoris*	Qinghai Lake, Qinghai, China	QHH-53	H3	CRX2466394	NGDC
195	*R.kukunoris*	Qinghai Lake, Qinghai, China	QHH-55	H3	CRX2466395	NGDC
196	*R.kukunoris*	Qinghai Lake, Qinghai, China	QHH-57	H3	CRX2466396	NGDC
197	*R.kukunoris*	Maktang, Jianzha, Qinghai, China	JZMKT-1	H1	CRX2464014	NGDC
198	*R.kukunoris*	Maktang, Jianzha, Qinghai, China	JZMKT-2	H2	CRX2464015	NGDC
199	*R.kukunoris*	Maktang, Jianzha, Qinghai, China	JZMKT-3	H1	CRX2464016	NGDC
200	*R.kukunoris*	Maktang, Jianzha, Qinghai, China	JZMKT-4	H3	CRX2464017	NGDC
201	*R.kukunoris*	Maktang, Jianzha, Qinghai, China	JZMKT-5	H4	CRX2464018	NGDC
202	*R.kukunoris*	Maktang, Jianzha, Qinghai, China	JZMKT-6	H5	CRX2464019	NGDC
203	*R.kukunoris*	Tongren, Qinghai, China	TR-1	H6	CRX2464020	NGDC
204	*R.kukunoris*	Tongren, Qinghai, China	TR-2	H7	CRX2464021	NGDC
205	*R.kukunoris*	Tongren, Qinghai, China	TR-3	H3	CRX2464022	NGDC
206	*R.kukunoris*	Tongren, Qinghai, China	TR-4	H7	CRX2464023	NGDC
207	*R.kukunoris*	Tongren, Qinghai, China	TR-5	H3	CRX2464024	NGDC
208	*R.kukunoris*	Tongren, Qinghai, China	TR-6	H8	CRX2464025	NGDC
209	*R.kukunoris*	Heri Town, Zeku, Qinghai, China	ZK-1	H3	CRX2464026	NGDC

Number	Speciesname	Locality	Samplenumber	Haplotype	Accession number	Database
210	*R.kukunoris*	Heri Town, Zeku, Qinghai, China	ZK-4	H3	CRX2464027	NGDC
211	*R.kukunoris*	Heri Town, Zeku, Qinghai, China	ZK-5	H3	CRX2464028	NGDC
212	*R.kukunoris*	Heri Town, Zeku, Qinghai, China	ZK-6	H3	CRX2464029	NGDC
213	*R.kukunoris*	Heri Town, Zeku, Qinghai, China	ZK-7	H3	CRX2464030	NGDC
214	*R.kukunoris*	Heri Town, Zeku, Qinghai, China	ZK-8	H3	CRX2464031	NGDC
215	*R.kukunoris*	Heri Town, Zeku, Qinghai, China	ZK-9	H3	CRX2464032	NGDC
216	*R.kukunoris*	Heri Town, Zeku, Qinghai, China	ZK-10	H3	CRX2464033	NGDC
217	*R.kukunoris*	Heri Town, Zeku, Qinghai, China	ZK-11	H3	CRX2464034	NGDC
218	*R.kukunoris*	Zequ Town, Zeku, Qinghai, China	ZKZQ-2	H3	CRX2464035	NGDC
219	*R.kukunoris*	Zequ Town, Zeku, Qinghai, China	ZKZQ-3	H3	CRX2464036	NGDC
220	*R.kukunoris*	Zequ Town, Zeku, Qinghai, China	ZKZQ-4	H3	CRX2464037	NGDC
221	*R.kukunoris*	Zequ Town, Zeku, Qinghai, China	ZKZQ-5	H3	CRX2464038	NGDC
222	*R.kukunoris*	Zequ Town, Zeku, Qinghai, China	ZKZQ-6	H3	CRX2464039	NGDC
223	*R.kukunoris*	Zequ Town, Zeku, Qinghai, China	ZKZQ-7	H9	CRX2464040	NGDC
224	*R.kukunoris*	Henan, Qinghai, China	HN-1	H3	CRX2464041	NGDC
225	*R.kukunoris*	Henan, Qinghai, China	HN-2	H3	CRX2464042	NGDC
226	*R.kukunoris*	Henan, Qinghai, China	HN-3	H3	CRX2464043	NGDC
227	*R.kukunoris*	Henan, Qinghai, China	HN-6	H9	CRX2464044	NGDC
228	*R.kukunoris*	Henan, Qinghai, China	HN-7	H3	CRX2464045	NGDC
229	*R.kukunoris*	Henan, Qinghai, China	HN-9	H3	CRX2464046	NGDC
230	*R.kukunoris*	Henan, Qinghai, China	HN-10	H8	CRX2464047	NGDC

Number	Speciesname	Locality	Samplenumber	Haplotype	Accession number	Database
231	*R.kukunoris*	Garang, Guide, Qinghai, China	GDGR-1	H3	CRX2464048	NGDC
232	*R.kukunoris*	Garang, Guide, Qinghai, China	GDGR-2	H2	CRX2464049	NGDC
233	*R.kukunoris*	Garang, Guide, Qinghai, China	GDGR-3	H2	CRX2464050	NGDC
234	*R.kukunoris*	Garang, Guide, Qinghai, China	GDGR-4	H3	CRX2464051	NGDC
235	*R.kukunoris*	Garang, Guide, Qinghai, China	GDGR-5	H8	CRX2464052	NGDC
236	*R.kukunoris*	Garang, Guide, Qinghai, China	GDGR-7	H8	CRX2464053	NGDC
237	*R.kukunoris*	Garang, Guide, Qinghai, China	GDGR-9	H1	CRX2464054	NGDC
238	*R.kukunoris*	Guomaying, Guinan, Qinghai, China	GNGMY-1	H5	CRX2464055	NGDC
239	*R.kukunoris*	Guomaying, Guinan, Qinghai, China	GNGMY-2	H3	CRX2464056	NGDC
240	*R.kukunoris*	Guomaying, Guinan, Qinghai, China	GNGMY-3	H3	CRX2464057	NGDC
241	*R.kukunoris*	Guomaying, Guinan, Qinghai, China	GNGMY-4	H10	CRX2464058	NGDC
242	*R.kukunoris*	Guomaying, Guinan, Qinghai, China	GNGMY-5	H5	CRX2464059	NGDC
243	*R.kukunoris*	Guomaying, Guinan, Qinghai, China	GNGMY-6	H1	CRX2464060	NGDC
244	*R.kukunoris*	Lajia Town, Maqin, Guoluo, Qinghai, China	LJ-1	H3	CRX2464061	NGDC
245	*R.kukunoris*	Lajia Town, Maqin, Guoluo, Qinghai, China	LJ-2	H3	CRX2464062	NGDC
246	*R.kukunoris*	Dongqinggou, Maqin, Guoluo, Qinghai, China	DQJ-2	H3	CRX2464063	NGDC
247	*R.kukunoris*	Dongqinggou, Maqin, Guoluo, Qinghai, China	DQJ-3	H3	CRX2464064	NGDC
248	*R.kukunoris*	Dongqinggou, Maqin, Guoluo, Qinghai, China	DQJ-5	H3	CRX2464065	NGDC

Number	Speciesname	Locality	Samplenumber	Haplotype	Accession number	Database
249	*R.kukunoris*	Dongqinggou, Maqin, Guoluo, Qinghai, China	DQJ-7	H3	CRX2464066	NGDC
250	*R.kukunoris*	Baiyu, Jiuzhi, Guoluo, Qinghai, China	JZBY-1	H3	CRX2464067	NGDC
251	*R.kukunoris*	Baiyu, Jiuzhi, Guoluo, Qinghai, China	JZBY-2	H3	CRX2464068	NGDC
252	*R.kukunoris*	Baiyu, Jiuzhi, Guoluo, Qinghai, China	JZBY-6	H11	CRX2464069	NGDC
253	*R.kukunoris*	Baiyu, Jiuzhi, Guoluo, Qinghai, China	JZBY-9	H12	CRX2464070	NGDC
254	*R.kukunoris*	Sailaitang, Banma, Guoluo, Qinghai, China	SLT-1	H3	CRX2466397	NGDC
255	*R.kukunoris*	Sailaitang, Banma, Guoluo, Qinghai, China	SLT-2	H3	CRX2466398	NGDC
256	*R.kukunoris*	Sailaitang, Banma, Guoluo, Qinghai, China	SLT-3	H1	CRX2466399	NGDC
257	*R.kukunoris*	Sailaitang, Banma, Guoluo, Qinghai, China	SLT-4	H3	CRX2466400	NGDC
258	*R.kukunoris*	Ganglong, Gande, Guoluo, Qinghai, China	GDGL-2	H3	CRX2464075	NGDC
259	*R.kukunoris*	Ganglong, Gande, Guoluo, Qinghai, China	GDGL-3	H3	CRX2464076	NGDC
260	*R.kukunoris*	Ganglong, Gande, Guoluo, Qinghai, China	GDGL-4	H3	CRX2464077	NGDC
261	*R.kukunoris*	Ganglong, Gande, Guoluo, Qinghai, China	GDGL-5	H3	CRX2464078	NGDC
262	*R.kukunoris*	Ganglong, Gande, Guoluo, Qinghai, China	GDGL-6	H3	CRX2464079	NGDC

Number	Speciesname	Locality	Samplenumber	Haplotype	Accession number	Database
263	*R.kukunoris*	Ganglong, Gande, Guoluo, Qinghai, China	GDGL-7	H8	CRX2464080	NGDC
264	*R.kukunoris*	Ganglong, Gande, Guoluo, Qinghai, China	GDGL-8	H3	CRX2464081	NGDC
265	*R.kukunoris*	Ganglong, Gande, Guoluo, Qinghai, China	GDGL-9	H3	CRX2464082	NGDC
